# Theorizing subjective responsibility at work: an agentic approach

**DOI:** 10.3389/fpsyg.2025.1548931

**Published:** 2025-07-10

**Authors:** Thomas Faurholt Jønsson, Maria Celeste Fasano

**Affiliations:** Department of Psychology and Behavioural Sciences, School of Business and Social Sciences, Aarhus University, Aarhus, Denmark

**Keywords:** objective responsibility, subjective responsibility in organizations, human agency theory, corporate social and environmental responsibility, power and competence

## Abstract

Along with an increased centrality of moral values and conduct in society and organizations, scholars’ interest in many forms of responsible organizational behaviors has proliferated. The present article intends to contribute to future organizational psychology by conceptualizing what subjective responsibility is and developing a general model of antecedents and consequences of subjective experience. We conducted a rapid literature review, with the purpose of mapping existing domains of responsibility, i.e., what does research in organizational psychology investigate responsibility *for*? There is much interdisciplinary literature about organizational level “objective” responsibility, e.g., Corporate Social Responsibility, but less about the subjective experience and dynamic nature of responsibility. Therefore, we specifically searched for theories and conceptualizations of responsibility as an organizational psychological, i.e., “subjective,” phenomenon. Our results confirm what other scholars have also previously observed, namely that theoretical groundwork centered on the psychological phenomenon of responsibility in organizations is rather rudimentary treated in the literature. This is unfortunate as thorough conceptualization and theory about phenomena generally form the basis for robust future research. Therefore, we integrate extant, theoretically underdeveloped concepts of subjective responsibility to reach a comprehensive definition of the phenomenon. Second, we develop a theoretical model that may be applicable to understand and hypothesize about organizational responsibility for various domains, such as responsibility for work outcomes or the environment. To cover the interplay between organizational structural factors and organizational members’ psychological level, we depart from the structure-agency metatheory. Thus, we assume that individuals subjectively regulate areas and degrees of responsibility in reciprocal interplays with structural properties. As outcomes, we focus on how responsible actions may be differently motivated. With the comprehensive conceptual development, we intend to lay the ground for a better understanding and measurement of the organizational psychological phenomena. Moreover, our theoretical model may be applied to support hypothesis development in the many different domains of responsibility, both with respect to organizational and personal antecedents and motivated responsible actions.

## Introduction

We could consider the start of the new millennium as the beginning of an “age of responsibility.” Not that responsibility itself is something new, but rather that the demand for responsibilities seems to have proliferated. Earlier on, responsible managers were valued for their fair and productive treatment of subordinates, which in turn fostered organizational cooperation and enhanced performance (e.g., Barnard, 1938; Smith, 1994; Winter, 1991). Responsible employees were desired based on a concern for production (e.g., Hackman and Oldham, 1976), organizational change (e.g., Morrison and Phelps, 1999), or extra-role behavior (e.g., Pearce and Gregersen, 1991). These areas remain important and have yet not been thoroughly investigated theoretically or empirically. However, in recent times, different social movements have demanded more responsibility for their respective areas of concern and rights. Business scandals, such as the case of Enron (e.g., Deakin and Konzelmann, 2004), and the financial, and banking crises of the late 2000’s and early 2010’s warranted financial responsibility. The environmental and climate crisis demands responsibility for nature and emissions of greenhouse gasses. The MeToo-movement urged for responsible treatment of women in positions of low power in virtually all industries. Such social developments and discourses are reflected in business sciences in that research on how organizations can take an active societal and environmental responsibility has proliferated. Organizational scholars have done much work in this area, but for the organizational psychologist, it remains somewhat unclear what responsibility really is from this perspective. Though some organizational psychology researchers have included variables measuring some aspects of subjective responsibility (e.g., Arain et al., 2019; Morrison and Phelps, 1999; Pearce and Gregersen, 1991), it has been done without resting upon a thorough theorization of what the psychological phenomenon behind fundamentally is and how it relates to other phenomena. Without such a theoretical grounding, empirical research risks to lack direction in, e.g., development of hypotheses, precise measurement, and cumulation of research results. Hence, this current situation risks holding back progress in our psychological knowledge about responsibility in organizations. The lack of comprehensive theory is recognized elsewhere: In their edited book on responsibility, Auhagen and Bierhoff (2002, p. 1) begin with the words: “What exactly is responsibility? This question cannot yet be answered comprehensively,” and they continue: It includes “many faces and facets. […] No overarching definition of the construct exists” (p. 3). Since Auhagen and Bierhoff’s bemoaning almost 25 years ago, little research has been made to grasp a unified framework about the essence of responsibility. A mentionable exception is Holdorf and Greenwald (2018), who performed a lexical analysis based on the Merriam-Webster dictionary and an interview study. These two studies showed that lay people understand responsibility as a construct with different meanings. This lack of an integrated theory about the psychology of responsibility, with subjective responsibility at the center, is unfortunate because it hinders the understanding of how individuals act and react to the many kinds of demands for responsibility in their organizational and social environments.

In this paper, we aim to contribute to an understanding of organizational responsibility by offering a psychological theoretical perspective on *subjective responsibility*. We attempt to achieve this in three steps corresponding to three main sections of the present article. The first step is to obtain an overview of relevant literature. Therefore, in the first section, we conduct a rapid literature review within organizational psychology and related organizational research fields using the Scopus and PsychINFO databases. The rapid review aims at (a) providing an overview of the amount of literature that develops concepts and theories about subjective responsibility, and (b) mapping the key themes in the literature on organizational responsibility. The next step is to apply the literature for conceptual and theoretical integration. Thus, in the second section, we try to respond to the calls for an overarching definition by Auhagen and Bierhoff (2002) and Holdorf and Greenwald (2018). Hence, we attempt to integrate relevant literature into an overarching definition of subjective responsibility. In the third section, we depart from the integrative definition and use the literature to develop a model based on human agency theory. The model intends to propose specific mechanisms in the psychological pathways between organizational responsibility and individuals experiencing themselves to be responsible and act accordingly. The first step in this model development is to develop theoretical propositions. Based on a structure-agency theoretical approach (Archer, 2003; Bandura, 2006) and the relevant literature about responsibility, we propose how organizational “objective” goals of responsibility and features of organizational structures may direct and facilitate (or undermine) human agency. Perceptions of organizational responsibilities and experiences of human agency may, in turn, affect how individuals regulate subjective responsibility. In the end, subjective responsibility may lead to different forms of motivated actions. We integrate the propositions into a comprehensive, general model. The last part ends with a discussion of the implications and limitations of the proposed integrative model.

## Overview of responsibility literature

### Literature search methods

To identify what and how much literature there exists about organizational psychological theory of responsibility, we first attempted to narrow our search to include only organizational psychology and its closest related fields. We began by searching in Scopus, a database renowned for its strength in the broad interdisciplinary domains of business and management, including organizational psychology. Following this, we expanded our search to PsychINFO, which is widely recognized as a leading database for research in psychology, including organizational psychology.

To ensure comprehensive coverage of research on responsibility in the workplace, we employed a multi-step search strategy across two domains: Applied Psychology and Business Management. To ensure a thorough representation of relevant literature within the subfield of Work and Organizational Psychology (WOP), we began by focusing our search on the domain of Applied Psychology domain. However, during the process, we recognized that many relevant journals within the broader organizational psychology field, including those related to management and organizational behavior, were underrepresented. Consequently, we expanded our search to include Business Management. This adjustment allowed us to capture key contributions from journals that focus on organizational behavior, leadership, and management, all of which are closely linked to the concept of responsibility at work. Additionally, we supplemented our search by including articles from General Psychology and Management that had been collected over the years from the first author’s literature database. This database, which includes studies gathered in an unstructured manner over time, provided valuable perspectives that might not have been fully captured through more formal, systematic search strategies. A summary of the search strategy, including the selection process and relevant journals, is provided in Supplementary Table 1.

### Identification of relevant journals in Scopus

We began by conducting a broad search within the Scopus database under the “psychology” category. The rationale behind this initial search was that Scopus does not specifically include a dedicated subfield for Work and Organizational Psychology (WOP). Our search focused on articles related to responsibility in the workplace, filtering for keywords related to work and organizational settings, such as *“work,” “organization,” “employee,” “leader,”* and *“business.”* We specifically targeted articles that mentioned the term *“responsibility,”* while excluding terms related to corporate social responsibility and accountability, as these were outside the scope of our study. The search results were further filtered by publication date, document type, and language (see Supplementary Table 1). From the total articles retrieved, journals were evaluated based on their inclusion in the Scimago database under the *Applied Psychology* category, as Scimago does not provide a distinct subcategory for Work and Organizational Psychology. This step allowed us to limit our search specifically to Applied Psychology, narrowing the scope of the review. Additionally, this step was essential to ensure the articles were published in reputable, peer-reviewed journals, filtering out non-peer-reviewed or lower-quality sources. While Scimago includes journals with varying impact factors, it remains a reliable tool for confirming the academic quality and credibility of journals, thereby ensuring that our review was based on high-quality research. Out of the 160 journals initially identified in Scopus, 48 were found to be listed in Scimago. From these, we assessed each journal for relevance to the research question. After screening, 21 journals were selected, all of which were deemed directly relevant to the study of responsibility at work, specifically within the realms of work psychology and organizational behavior (see the list of Journals in Supplementary Table 2). Journals that focused on unrelated areas, such as Developmental Psychology (e.g., responsible parenting and childcare), were excluded.

Furthermore, in order not to omit relevant articles within the “business management” domain, we subsequently used the Chartered Association of Business Schools (ABS) Journal Ranking List to identify further relevant journals. We focused on selecting journals from three specific ABS subfields: Organizational Studies, Work and Organizational Psychology / Organizational Behavior, Business Ethics, and Management (the latter only upper-level journals). Journals within these subfields were specifically chosen for their established reputation in the field of business management and their relevance to the study of responsibility in organizational and workplace contexts. Additionally, we included the Annual Review of Organizational Psychology and Organizational Behavior and Frontiers in Organizational Psychology, two high-impact journals in the field. We selected these journals due to their significant contributions to the literature on organizational behavior and psychology. By focusing on these relevant and well-regarded journals, we ensured that our review captured high-quality research while maintaining alignment with the core themes of responsibility, ethics, and organizational behavior (see the list of Journals in Supplementary Table 3).

### Article selection and screening in Scopus

We used Scopus to identify relevant articles published in these selected journals, ensuring that they addressed the theme of responsibility in organizational and work settings. After narrowing the selection to 21 relevant journals in applied psychology and the 116 selected business management journals, we identified a total of 72 articles in the *Applied Psychology* domain and 120 articles in the *Business Management* domain, all of which included “responsibility” in the title. This filtering process was intentionally designed to ensure that only articles with a substantial focus on responsibility were included.

The selected articles were screened, focusing on the abstracts and titles to ensure alignment with the research question on individuals’ subjective responsibility at work, as opposed to organizational level, objective conceptualizations of responsibility. Each article was classified as “yes,” “no,” or “maybe.” After screening, 147 articles were excluded for not meeting the relevance criteria, leaving 25 articles deemed relevant for inclusion in the study (16 within applied psychology, 9 within business management). Notably, none of the included articles were theoretical and specifically focused on subjective responsibility at work. The relevant articles were further analyzed and incorporated into the theoretical and empirical literature on the psychological aspects of responsibility in organizational settings, as detailed below. Additionally, a qualitative analysis was conducted on the articles that did not meet our criteria, revealing the main macro areas of the omitted literature, which are also discussed in the following section. Overall, the search results highlight a significant gap in organizational psychology research concerning conceptual and theory development about subjective responsibility.

### Supplementary search in PsycINFO

To ensure comprehensive coverage of literature on subjective responsibility, we conducted a supplementary search in the PsycINFO database, specifically narrowing the search to terms such as “felt responsibility,” “experienced responsibility,” and similar keywords in the title (see Supplementary Table 2). This focused search was done because PsycINFO is a specialized psychological database, offering in-depth access to psychological literature that may not be fully represented in broader, multidisciplinary databases like Scopus. While Scopus provides a comprehensive overview of the literature in both Applied Psychology and Business Management, it may not capture the full range of psychological constructs specifically related to subjective responsibility.

We further refined our search by restricting it to classifications within Applied Psychology, including categories such as “Professional Impairment,” “Organizational Psychology and Human Resources,” “Industrial and Organizational Psychology,” and others related to organizational behavior and human resources (see Supplementary Table 4). This adjustment allowed us to specifically focus on the psychological aspects of responsibility, which are central to our research.

By complementing the Scopus search with PsycINFO, we were able to capture a more comprehensive set of studies specifically focused on the psychological dimensions of responsibility, ensuring that our review was as inclusive as possible. This supplementary search allowed us to capture additional relevant studies, ultimately identifying 47 articles. After screening for relevance, 22 articles were deemed relevant and contributed valuable insights into the psychological aspects of responsibility in organizational and workplace settings. Of these 22 articles, four were already part of our database. The 22 articles were analyzed and integrated into the theoretical framework of our study.

### Search results: mapping key areas and identifying gaps

This rapid literature review allowed us to identify the key areas of research on subjective responsibility in organizational psychology and highlight the significant gaps that remain unaddressed. Our review revealed that while certain aspects of responsibility in the workplace have been widely discussed, there is a notable scarcity of research focusing on subjective responsibility in organizational contexts. We also identified that much of the existing literature centers on more objective or external forms of responsibility, leaving a critical space for further theoretical exploration of the subjective dimensions of responsibility in work settings.

Specifically, we found that the word “responsibility” is used in many ways in the field of WOP and related ones, and our analysis based on the literature search showed different research streams. Most often, the usage refers to “objective” phenomena, external to and beyond the subjective sphere of the individual, and they may be formalized, such as in a job description or a formal organizational hierarchy. Such “objective” understandings of responsibility include reference to a person’s duties to perform a certain role or it can be responsible to another person, often in the role of a supervisor or controller (MacLagan, 1983). In line with MacLagan (1983), we understand “objective responsibility” as features of individuals’ contextual levels that exist independently from the individuals’ subjective realm. Objective responsibility exists in the shape of formal arrangements or communications that can come from formal decisions about strategy and organizational goals or embedded in the formal organizational system. Functions and decisions about organizational responsibility may be executed and conveyed to organizational members by people in positions and roles authorized to deal with formal responsibilities and may meet the individual organizational member as managerial directions and formal or informal roles. See Table 1 placed at the end of the article.

**Table 1:** Distinguishing subjective from objective responsibility.

	Type of responsibility	
	Objective	Subjective
Location	Society and organization that form an external environment for individuals	Intra-individual
Form	Formalized or norm-based social expectations, for example formal job role or duties, goals and objectives	Experienced beliefs, a dynamic, contextually influenced psychological state
Origin	Formally decided, executed via management	Individually decided based on external demands and internal values
Regulation	Decision maker in the organization and in organizations’ environments, for example boards, leaders, lawmakers	Intra-individually regulated based on perceptions of external, objective demands for responsibility and personal power and competence
Control mechanisms	External control mechanisms, embedded in, for example, accountability systems or other formal monitoring and evaluation	Internal control mechanisms based on experienced responsibility areas and degree
Example; work responsibility	Formal work process design with work roles and goals	Individuals’ own experienced responsibility for tasks related to perceived work role
Example; CSER	Organizational strategic CSER goals and practices, leaders’ directions about how to act with respect to CSER	Individuals’ own experienced responsibility for CSER actions related to perception of CSER

The largest stream of research that includes responsibility as a personal, experienced phenomenon stems from Hackman and Oldham (1976) Job Characteristics Theory. Without further conceptualization of responsibility, they proposed responsibility for the outcomes of work (together with meaningfulness and knowledge about work) to be a mediator between job characteristics and outcomes hereof. However, all three mediating psychological states were mostly neglected in subsequent research. Thus, 25 years later, a meta-analysis by Behson et al. (2000) noticed that job design researchers still generally omitted the three mediational variables in their research.

A smaller stream of responsibility in Organizational Citizenship Behavior (OCB) follows Morrison and Phelps (1999) and focuses on “felt responsibility for constructive organizational change” as a mediator between structural factors and OCB outcomes (e.g., Fuller et al., 2006).

Other researchers see responsibility as related to power (ab)use. For example, Winter and Barenbaum (1985) investigate the Need for Power and, here, felt responsibility operates as an inhibiting factor between high power need and its (abusive) behavioral consequences. However, the Need for Power is the focal variable, and responsibility plays only a “supporting role” in McClelland and colleagues’ needs theory (McClelland, 1987). The role of responsibility in using power over others has been further investigated, for example, by De Wit et al. (2017).

We also find the term “responsibility” used in literature about “accountability systems,” which are organizational arrangements aimed to create documentation and rewards (or sanctions) for compliance with formal criteria of responsibility. Accounts are made to keep people accountable for their actions (Dose and Klimoski, 1995).

In the new millennium, the areas of responsibility expand beyond work processes. For example, a stream of research literature investigates situations in which employees have family responsibilities, for example caring for an elderly family member or child. This stream of research has several aims: to develop “family responsive workplaces,” to investigate discrimination against employees with special family responsibilities, or to investigate the consequences of such demanding responsibilities (e.g., Dickson, 2008).

Responsibility is also often used at organizational levels, particularly in research about Corporate Social (and Environmental) Responsibility and Business Ethics. Aguinis (2011) sums the field up and defines organizational responsibility “as context-specific organizational actions and policies that take into account stakeholders’ expectations and the triple bottom line of economic, social, and environmental performance” (p. 855). In this case, responsibility is not a subjective property of an individual person but something an organization pursues. However, focusing on the individual level, research tends to investigate what outcomes there are for the employee engaging in CSR-activities (Glavas, 2016). While Glavas (2016) review offers several fine proposals for individual level CSR-mechanisms, none of them expresses that the individual employee may subjectively choose to feel responsible and be motivated by acting responsibly. A new branch in this area of research is “digital responsibility,” which is aimed at promoting a sustainable and fair digital society (Trier et al., 2023). A further development from the organizational responsibility field is Responsible Leadership, which is when business leaders provide collective value to stakeholders, including employees, customers, society, and the environment through win-win solutions, ethical decision-making, and actions (Pathak and Jha, 2024). Similarly, a subfield of CSR and Responsible Leadership is the Responsible Innovation area which takes a stakeholder perspective on innovations (e.g., Bacq and Aguilera, 2022).

All these approaches to responsibility deal with external, objective, organizational, and societal level understandings of responsibility as a moral endeavor to safeguard moral stakeholder interests and values.

What we are mostly interested in here is the subjective responsibility that people experience and regulate psychologically. People need to take and feel the responsibility to and by themselves to intrinsically commit and act responsibly, which can be to align with accountability systems’ criteria, act toward CSER goals, within Responsible Leadership or not, etc. These “outer,” “objective” kinds of responsibility all need an “inner,” “subjective” form of responsibility to realize their goals. Though we found some WOP research on “felt” or “sense of” responsibility, we did not find a thorough theoretical elaboration of this phenomenon in organizational psychology. We attempt to do so below.

## An integrative definition of subjective responsibility

### Subjective responsibility

Conceptual research on responsibility has played a relatively rudimentary role in psychology, and there is little consensus regarding the concept (Auhagen and Bierhoff, 2002; Holdorf and Greenwald, 2018). Here, we expand on how we derive the definition from extant literature. In the section below, we review literature that defines or measures responsibility in organizations and apply it to elaborate on the elements of the present definition of responsibility. We forestall the conceptual review of the literature below and define the subjective phenomenon of responsibility as *the personal experience of feeling obligated to take initiative or to take precautions to ensure success and avoid errors concerning a specific valuable target.*

### Subjective experience

First, responsibility may be defined externally or “objectively,” such as, for example, the formal duties of a work role or informal social norms (Kulik et al., 1987; MacLagan, 1983). However, this study focuses on the *subjective* aspect of responsibility. Therefore, we treat it as something inferred, which may emerge as a conscious experience or remain relatively unexperienced, as a preconscious intuition. This is in line with a number, if not most, of psychology scholars who touch upon the concept. For instance, in his attribution theoretical approach, Weiner (1993, 1995b) views responsibility as a social cognition about the judgment of others. This is similar to Lenk (1992), who states that responsibility is essentially always viewed by *someone* who judges responsibility (Lauermann and Karabenick, 2011). Others underline the subjectivity of the concept with words such as *felt* or *feeling of* (e.g., Hackman and Oldham, 1976; Morrison and Phelps, 1999; Nowell and Boyd, 2014; Pearce and Gregersen, 1991) or *sense* of responsibility (e.g., Boyd and Nowell, 2017; Lauermann and Karabenick, 2013). Some understand responsibility as a *belief*, i.e., a cognitive construct (Morrison and Phelps, 1999) or a *psychological state* (e.g., Hackman and Oldham, 1976; Punzo et al., 2019). Hackman and Oldham (1976) view “felt responsibility” as the belief that one is personally accountable for the work outcomes. Their understanding is clearly reflected in one of the few responsibility items in their Job Diagnostic Survey, which inquires whether the respondent feels a “high degree of *personal* responsibility” (Hackman and Oldham, 1975, Section 3, item 8, original underlining). We note that by defining responsibility as a subjective and perceptional phenomenon, we are distinguishing it from the type of responsibility associated with accountability systems (Dose and Klimoski, 1995). By using the word subjective instead of “felt” we wish to distinguish from externally defined, “objective” responsibility and, furthermore, we aim to underline that it is not merely a perception, but also a phenomenon with intrapsychic, subjective dynamics involved. Finally, we acknowledge that the aforementioned perspectives on subjective responsibility—as cognition, something inferred, a perception, judgment, or belief—align with the view of responsibility as a psychological state, that is, a dynamic, changeable, and situational phenomenon. While we fully agree with this conceptualization, we also remain open to the possibility that certain personality traits may facilitate the experience of responsibility.

### Obligation

One of the most prevalent understandings of responsibility is that it contains a sense of duty to fulfill obligations and moral principles related to the particular responsibility *in situ*. This quality of *oughtness* makes responsibility a type of moral concept (Holdorf and Greenwald, 2018). For example, Morrison and Phelps (1999) call it a “personal obligation” (p. 1999). Some researchers seem to equate obligation and responsibility. Thus, Liu et al. (2021) write’ felt obligations is the mental state of employees, who are actively responsible for their work results’ (p. 184). They applied Eisenberger et al.’s (2001) concept of felt obligation to reflect a subjective mediational mechanism between (objective) CSR and voice behavior. A felt obligation is the belief that one should care for and help the organization reach its goals and achieve wellbeing. It is a reciprocation of perceived organizational support (Eisenberger et al., 2001). Dose and Klimoski (1995) define it as a perceived obligation for a situation or event (p. 36). Boyd and Nowell (2017) also emphasize the duty-dimension of responsibility and define a “Sense of Community Responsibility as a feeling of duty or obligation to protect and enhance the wellbeing of a group and its members” (p. 213). They see responsibility as the key to understanding why people sometimes sacrifice their own individual needs for the benefit of their community. We also find this line of thinking with respect to extra-role behavior. Pearce and Gregersen (1991) propose that responsibility includes a motive to serve something other than oneself, and that responsibility is one out of more motives for prosocial, altruistic action. They apply this understanding to Organizational Psychology and propose that responsibility for the organization can explain why employees choose to engage in extra-role behaviors rather than merely focus on their own formally prescribed jobs. In other words, responsibility encompasses a moral obligation of something that goes beyond the mere maximization of egoistic gains (the special case of self-responsibility excluded). Winter (1991) calls it an inner obligation to do what is right, which makes the person dependable for others. This conceptualization of responsibility also implies that responsibility is to perform a duty within the moral boundaries of that role. In Winter’s perspective, responsibility is an ability to inhibit impulses and desires, so that a leader does not abuse leadership power to serve egoistic goals. Thereby, a responsible leader is dependable and predictable to others.

### Action imperative

Responsibility is not merely a perception of duties and obligations. It also pertains to action. Responsibility entails an imperative to act to safeguard and take care of the target of responsibility; responsibility is about responding actively to a subject matter and *ex-ante* an event. This theoretical point is shared among several scholars. It is perhaps most emphasized in the conceptualization of responsibility by Morrison and Phelps (1999), who see responsibility as a motive behind taking initiatives, i.e., to start an action. According to them, responsibility comprises the decision to respond actively, even in light of risks associated with actions deemed responsible. They support it by showing that responsibility for constructive organizational change is positively related to taking charge at work (Morrison and Phelps, 1999). Their measurement of “felt responsibility” has been adapted and applied in research on environmental responsibility (e.g., Lu et al., 2022; Punzo et al., 2019). In this line of research, responsibility is understood as a psychological state that turns values into action. MacLagan (1983) describes responsibility as “one is deemed to be the cause of” something to happen (p. 411), and Winter and Barenbaum (1985) in-depth analyses of TAT stories among highly responsible persons with high power need revealed that they had a strong sense of obligation to act out of a rule, principle, or duty. This is inspired by, *inter alia*, Chester I. Barnard, who understands responsibility as the action side of morality. He conceives responsibility as living out moral beliefs and acting in accordance with moral values. It includes coping adequately with moral dilemmas. This action imperative can also be found in Weiner (1995b), who provides experimental evidence that people punish those who make no effort more than those who make an effort. He explains that people attribute responsibility for not making an effort and therefore blame such a person. Overall, responsibility necessitates thinking forward, anticipating the consequences of various possible actions, and acting in a timely manner. The opposite of taking initiative (i.e., passivity, negligence, and ignorance) is irresponsible (Holdorf and Greenwald, 2018; Weiner, 1995b). This initiative and action element is in line with most other conceptualizations.

### Consequences—both success and failure

Responsibility aims toward securing beneficial outcomes and preventing negative consequences. Responsibility is *for* something (Lauermann and Karabenick, 2011; Lenk, 1992), and in this sense, responsibility is tied to a motive, such as a goal, value, or interest. For example, Hackman and Oldham (1976) focus on responsibility for work outcomes; Morrison and Phelps (1999) deal with responsibility for organizational change; Boyd and Nowell (2017) explore prosocial responsibility for the community. The whole CSER and Business Ethics literature focuses on responsibility for CSER values and other ethical values (e.g., Punzo et al., 2019).

To have responsibility for something means that an agent is bound to take care of a given area; therefore, success or failure within the area will elicit feelings of pride or guilt (Hackman and Oldham, 1976). Indeed, Lauermann (2014) claims that the motive to prevent or promote certain outcomes is what distinguishes responsibility from constructs such as self-efficacy and internal locus of control. Weiner (1993, 1995b) focuses on the prevention of negative outcomes, and he views responsibility as a social cognition about the judgment of others in situations of failure. In his view, responsibility and blame are two sides of the same coin. Diamond and Allcorn (1984) define it in terms of owning and taking the blame for the consequences of one’s actions. In these senses, responsibility is also “blameworthiness.” This failure emphasis can distinctively be found in Jackson et al.'s (1993) definition of “production responsibility,” which is responsibility for hindering errors and damage in the production process. While blame is a likely result of perceived failure, others focus on responsibility for success and concomitant emotions of pride and satisfaction (Hackman and Oldham, 1976), which represents the positive side of responsibility.

In sum, based on our review of how responsibility has been understood in the extant theory and research, we have identified the elements of subjectivity, obligation/duty, action imperative, and consequences. Tying these elements together, we suggest that *subjective responsibility is the experience of an obligation to take the initiative to act or to take precautions to ensure success and avoid errors with respect to a specific target of value.*

## Developing an agentic responsibility model

Having proposed an integrative conceptualization of subjective responsibility, we intend to develop a model of antecedents and consequences of it. Since we find responsibility to be an action-oriented phenomenon and a mental state that activates a person, we propose that responsibility plays a role in human agency, i.e., persons’ proactive actions intended to achieve goals, realize values, or safeguard interests. We elaborate on this below.

Bandura (2006, 2018) defines human agency as intentionally influencing one’s functioning and life circumstances (p. 164), including using human capacities to act proactively, self-organize, self-regulate, and self-reflect. From a human agency perspective, people not only react to environmental stimuli; they also shape the environment and choose whether to respond to an environmental stimulus. According to Archer (2002, 2003), agency is shaped by a structure of conditioning factors that enable or constrain actions in the person’s environment. In turn, an action may either reinforce or alter the environmental structure, depending on the intentions and personal powers of the agent (Archer, 2003). The person may also either be the same or change depending on whether the action is successful or a failure (Bandura, 2006). In this way, agency theory assumes a reciprocal causality between person and environment, between agency and structure. To underline the importance of acting, Bandura (2006) includes behavior *per se* as a third element in the reciprocal triangle of the person (the agent) and environment (the structure). These three dimensions co-cause each other in a person’s functioning and development (Bandura, 2006).

To the extent that subjective responsibility is an agentic property, it is part of the same reciprocal triangle and exists within the same agentic modes. Firstly, subjective responsibility is an intrapsychic phenomenon, thus belonging to the person-element. Consequently, responsibility may be reciprocally affected by behavior and environmental factors. Hence, subjective responsibility may change as behavior succeeds or fails to act responsibly, i.e., to safeguard the goals, values, or interests that may be embedded in the perceptions of responsibility at hand. In other words, if behavior takes responsibility by taking care of what responsibility is for, it will uphold the person’s subjective responsibility. If not, it may not only induce feelings of guilt and shame but may also reduce the feeling of responsibility in the future. Bandura showed mechanisms of how morality could be reduced through moral disengagement (Bandura, 2002), and similar yet other mechanisms may be in play when people regulate their responsibility. Based on these notions, we intend to develop an agency model of responsibility that incorporates structural properties, personal properties, and resulting actions that feedback on structure and person. We will focus on structural and personal properties and build propositions about the interrelationships.

### Structural factors

As noticed above, the concept of structure designates the external, conditioning factors that enable or constrain a person’s agency, including enable or constrain what to be responsible for and how much. Specifically, structural factors may both direct individuals’ choice of what to feel responsible *for* and affect their regulation of the *degree* of subjective responsibility. These two structural properties are treated in the following. First, we focus on how organizational structural features may direct what areas organizational members find themselves to be responsible for. Thereafter, we look at structural properties that may affect individuals’ regulation of the degree of subjective responsibility.

### Responsibility directing factors

Responsibility directing structural features are found in, for example, formulations of strategies (e.g., CSER or ethical goals), formal managerial communications, formal job descriptions as well as informally in culturally anchored communication and behaviors that provide information about priorities, and responsibilities (cf. Card, 2005). For example, formal and informal work roles allocate tasks and duties to employees, and these tasks and duties may be performed with or without feeling responsible for successful goal achievement. In this sense, organizational work role systems contribute to a formal, “objective” responsibility structure that may instigate organizational members’ acceptance of responsibility. Organizations may also have accountability systems aimed at making their members act responsibly as specified in these systems, and organizational members may follow the guidelines and document their actions with or without any intrinsic feelings of responsibility (Dose and Klimoski, 1995). The enacted responsibility may, on the other hand, also change organizations’ role systems (e.g., job crafting, see Tims and Bakker, 2010) or accountability systems – often in cases of failure to achieve responsible behavior. CSER strategies and goals are also examples of structural factors aiming to enhance behavior directed toward employees taking responsibility for these values and goals (Glavas, 2016).

Co-workers and team level phenomena may also constitute an influential environment for directing responsibility. For example, Wang et al. (2022) found that team level safety stressors had a negative cross-level effect on employees’ felt safety for workplace safety, and Freitas et al. (2019) found that coworkers’ safety-related behaviors and attitudes were positively related to felt responsibility for transferring learned safety knowledge to be used in their jobs. Freitas et al. (2019) explain how employees’ social information processing (Salancik and Pfeffer, 1978) may aid employees to identify and understand social expectations about their responsibility. Moreover, social exchange theory (e.g., Cropanzano et al., 2017) may explain how subjective responsibility may be directed by others’ as a reciprocation of perceived care and support (Freitas et al., 2019).

To the employee, leadership is an environmental factor, and leadership also defines and directs what employees should be responsible for. Hence different forms of leadership emphasize different areas that the employee is responsible for. For example, task-oriented leadership delegates and instructs about work role responsibilities, and change-oriented leadership attempts to make people responsible for implementing organizational change (Yukl et al., 2002). Another example: Responsible Leadership conveys CSER and ethical responsibilities to the employees (Pathak and Jha, 2024; Voegtlin, 2016).

However, these structural factors may exist without all people recognizing them. Perception is a dynamic process that is based on both formal and informal structural features as well as individual activities and diversity of information (Rauterberg, 1999). Thus, individuals may vary in whether and how they perceive objective and organizational responsibilities, and without perceiving what responsibilities the job and organization undertake, it may have no psychological effects on the people inhabiting the organization. Formal and informal (e.g., co-worker and managerial communication about responsibilities) structural sources of information may be integrated in interpreting what to be responsible for (cf. Rousseau, 1995). In organizational psychology, it is well known that employees’ understanding of their job role affects several psychological constructs such as job satisfaction and performance. For example, role perception, role clarity, and role conflicts are well-established constructs that tap into how people perceive objective role responsibilities (King et al., 1990). Role identity theory provides further insights in how dynamic, organizational, social, and personal processes form an individual’s perception of his or her role and how this perception may develop (Sluss et al., 2011).

**Figure 1:**
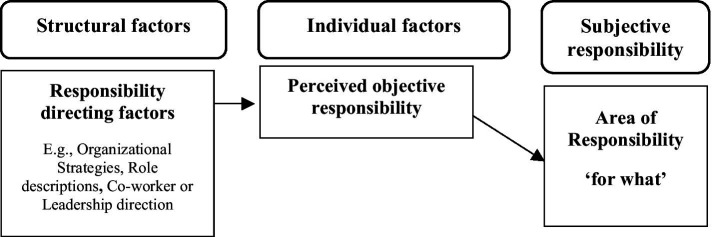
Structural direction of responsibility and the mediating role of individual perception. The figure distinguishes between structural and individual factors of responsibility. It illustrates how structural factors may direct what an individual subjectively feels responsible for. This direction may be mediated by the individual’s perception and interpretation of these structural factors.

Following the same logic, Glavas and Kelley (2014) broke with research that only measured CSR objectively and at the level of organizations and started to measure individual employees’ *perceived CSR*. Similarly, for organizational accountability systems to work, they require employees to perceive that they are accountable and must be ready to account for their actions and know of the rewards or sanctions (Hall et al., 2017). These notions lead us to the following propositions, which can also be seen in Figure 1.

**Figure 2:**
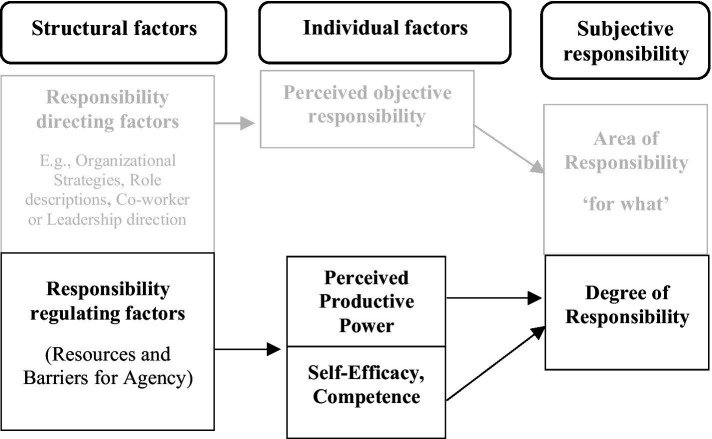
Regulation of subjective responsibility through perceived power and self-efficacy. The figure extends Figure 1 by including propositions 2 and 3. The figure shows how structural properties may affect perceptions of power and self-efficacy, which may, in turn, regulate the degree to which individuals experience responsibility.

*Proposition 1*: Structural factors such as organizational goals and strategies, co-worker and leadership behavior, or role descriptions, encompass directions about what organizational members should be responsible for, but individual perceptions hereof mediate the impact on what people subjectively experience to be responsible for.

### Responsibility regulating factors

Aside from structural properties that direct and prioritize organizational responsibilities, structural factors may also constitute resources and opportunities that intrinsically enhance (or diminish) the degree of subjective responsibility. These refer to factors that enhance independent (or interdependent), self-directed agentic actions, such as those found in employee empowerment theory (Dennerlein and Kirkman, 2023), and factors that enhance efficacy beliefs (Bandura, 2006; Hannah et al., 2008). Thus, we attend to structural factors that may facilitate agentic behavior by affording the agent the ability to utilize his or her powers and competencies to act successfully (Bandura, 2006). We propose that the levels of self-efficacy and power that the structure affords will regulate the degree of responsibility that a person will take on so that responsibility aligns with the structural affordances (and hindrances). It means that people perceive and assess their environment, the goals and challenges as well as resources and opportunities and regulate their level of subjective responsibility to fit with a tradeoff between hindrances and opportunities to act responsibly and safeguard the values or interests that responsibility is for. This regulation serves to enhance the probability of successfully taking responsibility and avoiding failing to live up to one’s responsibility. We dive deeper into how literature, in different ways, has assumed and tested the relationship between responsibility on the one hand and power and self-efficacy on the other.

#### Structure and productive power

In essence, power is the capacity to affect behavior, decisions, and beliefs in a desired direction (cf. Lukes, 2005). Personal power is the ability to autonomously change or willfully uphold the social structure. The power concept is thus a *productive* “power to” act according to intention (Archer, 2002). These theoretical approaches differ from “power over,” which is a relational type of power, in which one party dominates another. The “power to” approach is most prominent in organizational psychology in the Employee Empowerment tradition, dating back to pioneering empowerment scholar Kanter (1979) theory about power as productive. Two interrelated constructs form the productive power of empowerment, namely Self-determination and Impact. The former is autonomy and freedom to act independently at work, and the latter is influence on the wider organization, for example, through employee participation in decision-making (Spreitzer, 1995). Empirically, the two forms of productive power, autonomy, and organizational impact, are highly interrelated, also more interrelated than the other dimensions (Seibert et al., 2011).

Autonomy is also a key construct in Self-Determination Theory; in this theory, autonomy is “self-governance, or rule by the self” (Ryan and Deci, 2006, p. 1562). It means that an agent is fully contemplated about, identifies with, and endorses an autonomous act as opposed to behavior based on external demands or inner impulses. Within the field of I/O psychology, autonomy pertains to doing a job, including conditions of immediate relevance for doing that. The definition of autonomy by Hackman and Oldham (1976) is ubiquitous in autonomy literature. They view job autonomy as “[t]he degree to which the job provides substantial freedom, independence, and discretion to the individual in scheduling the work and in determining the procedures to be used in carrying it out” (p. 528).

While autonomy deals with power over one’s actions, power may also direct outwards of one’s organizational environment. Kanter (1977, 1981) understood organizational power as arrangements that provide access to information, resources, and support, as well as opportunities to advance, develop, and innovate. Her pioneering theory has widely influenced later structural empowerment research, such as Spreitzer’s (1996) work showing that empowerment is enhanced in work units providing role clarity, sociopolitical support, access to information and a participative climate as well as having a manager with a large span of control. Similarly, Boxall (2019) review practices under the label “High-involvement Work Practices and Systems” that focus on how organizations afford their members with power, information, rewards and knowledge/skills, which would result in higher levels of employee involvement, skill utilization and performance. Likewise, “participative” or “democratic” organizational structures has inbuilt sharing power in tactical and strategic areas, which encompasses decision power on mid- and long-term decisions such as staffing, personnel policies, process improvement, and purchases, as well as strategy, financial decisions, and organizational restructuring (Jeppesen et al., 2011; Weber et al., 2009). Employee involving and power sharing practices may also be stimulated via organizational cultures and climates. Values and tacit norms may affect perceptions, judgments and behavior with respect to how employees and managers cooperate in an involving way. For example, Weber et al. (2009) coined the socio-moral climate to designate how employees are involved in communication, cooperation and conflict in a participative, productive and egalitarian way. And Bosak et al. (2017) develop an Employee Involvement Climate, a shared perception of how an organization prioritizes and enables employee involvement in organizational decisions. The kind of power that is at play in employee involvement practices is productive in the sense that shared influence on the shared environment may lead to mutually considerate decisions and win-win solutions (Tjosvold, 1998). By implication, the power concept is also “power with” [as described by Mary Parker Follett, see Wheelock and Callahan (2006)]. This kind of productive power may also be what Archer (2002) understands when she describes power to be a possible collective property.

Managerial practices and several leadership styles play an important role in affecting organizational members’ feelings of autonomy and power (Llorente-Alonso et al., 2024). Participative and empowering leadership styles aims explicitly at providing autonomy and influence to the employees, but transformational leadership also afford employees voice and influence via the subdimensions “individual consideration” and “intellectual stimulation” (Schermuly et al., 2022). So-called new genre leadership (authentic, servant, ethical leadership) also prescribe involving employees in true dialogues and influence sharing (Llorente-Alonso et al., 2024; van Dierendonck, 2011). And opportunity-enhancing practices, a subcategory of High Performance Management Practices, aim at involving employees in decision-making and goal-setting (Chamberlin et al., 2018). All in all, organizational structure and arrangements as well as leadership may provide organizational members with experiences of power. In the following paragraphs, we will further theoretically investigate the relationship between power and responsibility.

#### Power and responsibility

In the extant theory and empirical research, we find several notions about power, influence, and control to be affecting responsibility. Weiner (1995b) argues that responsibility is “intimately linked with freedom and choice […and…] necessitates internal and controllable causality” (pp. 7–8). He emphasizes that responsibility and controllability are different phenomena but proposes that perceptions of controllability lead to judgments of responsibility. Here, controllability includes both personally being in control of one’s own actions and the degree of control of causal factors of a given situation. Hackman and Oldham's (1976) Job Characteristics Theory hypothesizes that job autonomy enhances employees’ experience of responsibility for the outcomes of their work. They explain that autonomy allows the employee to perform the job based on his or her own efforts, initiatives, and choices, rather than managerial and organizational control arrangements. In other words, the work outcome is up to the employee because of job autonomy. While many studies have tested the relationships between job characteristics and outcomes, such as motivation and performance, relatively few studies have paid attention to autonomy and responsibility (Behson et al., 2000; Fried and Ferris, 1987; Humphrey et al., 2007; Renn and Vandenberg, 1995). Four decades after the development of Job Characteristic Theory, a meta-analysis by Humphrey et al. (2007) showed that job autonomy was positively associated with responsibility for the outcome of work in 23 prior studies.

In discussions about accountability systems in organizations, it is often underlined that the difference between (external) accountability and subjectively experienced responsibility is that the former comes with a great deal of external, organizational control compared to the latter’s employee control and influence (Dose and Klimoski, 1995). In other words, responsibility may be based on trust and employee control. MacLagan (1983) discusses the difference between externally imposed and internally felt responsibility as well. In his view, the former pertains to formally prescribed duties and assignments, which are part of the formal bureaucratic organizational control system. Compared to the concept of accountability, MacLagan’s scope is broader and includes the total organizational setup of organizational control and instrumentalization of employees. This form of externally controlled formulations of responsibility differs from what kind of responsibility people would feel inside themselves, also in that the latter builds on respect for the single individual and autonomous development of awareness. According to MacLagan (1983), leaders should support employees’ feelings of responsibility for their own and organizational goals, for example, through employee participation in group goal setting (Erez et al., 1993). MacLagan (1983) stresses that such participation needs to involve genuine employee influence in order to elicit feelings of responsibility. We may also interpret the results from Morrison and Phelps (1999) as an empirical link between power and responsibility. Their analyses showed that felt responsibility and managerial position correlated significantly. A higher management position provides more power to a person, and it is this power that elicits perceptions of higher responsibility for work and the organization in general.

All in all, the reviewed research links responsibility to different elements of power (e.g., job autonomy, managerial position, control of the situation). Following this integrative view of the research, we propose that experiences of power may predict how responsible a person feels.

*Proposition 2*: Structural factors, such as structural empowerment, high involving work practices, empowering leadership, and participative climate, influence perceptions of productive power (i.e., job autonomy and organizational influence), which, in turn, affect regulation of subjective responsibility.

#### Structure, self-efficacy, and responsibility

Similar to the case of power, structural factors may also elicit experiences of competence—or, in Bandura’s terms, “self-efficacy” (Bandura, 2006; Spreitzer, 1995). Leadership for learning (Lundqvist et al., 2023), organizational learning culture (Xie, 2019), and organizational arrangements and practices (Garavan et al., 2015) that aim to enhance competence levels and thereby also likely impact organizational members’ self-efficacy or competence beliefs. According to Bandura’s Social Cognitive Theory, there is a triadic interaction between the social structure, self-efficacy, and behavior. Structural factors may facilitate or impede an individual’s mastery experiences, vicarious learning, and social support. All which affect self-efficacy (Bandura, 2006). Structural empowerment theory proposes that structural factors, such as organizational factors, leadership style, reward systems and job design form opportunities and hindrances for developing high self-efficacy (Conger and Kanungo, 1988). We understand self-efficacy in light of competence as the capacity to perform intended actions by manipulating objects via tools to obtain the desired goal of a task. Competencies may be understood as a cognitive and behavioral repertoire that a person can apply to predict, control, and cope with difficult and unpredictable challenges (Morrison et al., 2005). Belief in one’s own competence may thus provide an important resource for successfully undertaking responsibility, and hence for individuals’ upregulation of subjective of responsibility.

This is in line with the scarce research on the area, which indicate that the experience of competence may drive feelings of responsibility. Lauermann (2014) shows that teachers believe that competencies are important factors of their own experienced responsibility. The competencies that the teachers mention pertain to living up to the responsibility by means of their education, specialization, and other skills. Scholars share this understanding; for example, Diamond and Allcorn (1984) propose that responsibility requires mastery of one’s work. Research also uses Bandura’s socio-cognitive and learning theory about self-efficacy, i.e., beliefs in the capacity to act as intended. This capacity is based on past mastery experiences, role models accomplishing challenging tasks, encouragement, and calm emotions (Bandura, 1997). As such, competencies are a great part of the capacity to act, and some empowerment researchers consider competence and self-efficacy to be the same phenomenon (Spreitzer, 1995). In support of the notion, Morrison and Phelps (1999) found positive correlations between felt responsibility for constructive organizational change and general self-efficacy and expert power, which is interpersonal power based on *others’* beliefs about one’s competencies (French et al., 1959). Weiner (1995b) found that when people perceive that another person’s performance is based on ability and best efforts, rather than effortlessness and coincidence, they reward that performance higher. He explains that ability and effort determine the attribution of responsibility to the person, which then guides whether to reward that person or not for a performance. Lauermann and Karabenick (2013) found that teachers’ responsibility and teachers’ self-efficacy correlated moderately to highly. Feelings of competence are central to the concept of self-efficacy (Spreitzer, 1995). All in all, theoretical notions and empirical correlations suggest a link between the structural facilitation of self-efficacy or perceived competence and the degree of subjective responsibility.

*Proposition 3*: Structural factors, such as structural empowerment, leadership for learning, learning culture and competence development practices, influence self-efficacy, which, in turn, affects individuals’ regulations of subjective responsibility.

### Personal motives and responsibility

Besides the structural factors that are external to the individual, internalized values, goals, and motives of the individual may influence the choice of what to accept or reject responsibility for.

Since responsibility is always for something, personal values, and motives may affect what people wish to be responsible for. Values and motives are inherently related to responsibility, which is reflected in that Barnard (1938) as well as Punzo et al. (2019) understand responsibility as a psychological state that enacts one’s values in action. We follow this notion. If something is important to us, we are simply more compelled to take responsibility for it. If we focus on responsibility for work outcomes (cf. Hackman and Oldham, 1976), constructs such as job involvement or perceived meaningfulness of the job may direct an organizational member toward responsibility for the job. In his review of CSR in organizational psychology, Glavas (2016) also proposes a number of values and motives to be mechanisms that turn structural CSR initiatives into positive outcomes, such as the alignment of these values and motives with the CSR-motive making CSR purposeful and compelling.

Prosocial motives are also mentioned as responsibility may often demand some sort of self-sacrifice (Boyd and Nowell, 2017). Perceptions of organizational justice and support may also stimulate motives to accept and take organizational responsibility as a reciprocation (Eisenberger et al., 1990; Glavas, 2016). A clear illustration of the point that values and motives may direct what people subjectively choose to be responsible for is the study by Hahn et al. (2024). They showed employees’ disagreement with organizational CSR goals created tensions. People reacted by engaging in bottom-up initiatives aligned with their own environmental goals or negotiating the priorities between different initiatives. Their study also showed that misaligned personal values and motives downregulated other employees’ engagement, and the latter suggests that values and motives also play a role when regulating the degree of subjective responsibility. The motivating potential in values and motives may also depend on how internalized or how prioritized the values are (Schwarz, 2012) or how committed the individual is to goals and motives (Meyer et al., 2004).

*Proposition 4*: The more internalized values and motives are, the more these values will determine choices to accept responsibility for things aligned with these values and motives.

In sum, although structural factors provide the foundation for responsibility, individual factors—such as perceived power, self-efficacy, competence, and personal motives—affect the degree to which these structural cues are internalized and acted upon.

### Motivation

The key scope of subjective responsibility is to act responsibly. Whether it is task performance (Hackman and Oldham, 1976), extra-role performance (Pearce and Gregersen, 1991), organizational change implementation (Fuller et al., 2006), or CSR behavior (Mallory et al., 2021). However, how may responsible action be motivated? We attempt to deal with this issue and propose that high levels of responsibility will lead to intrinsically motivated as well as high levels of extrinsically motivated actions.

When viewing responsibility from an agency perspective, responsibility may operate as a “standard” in a goal-oriented agency. A standard is a goal that is used to compare a present state or achievement (informed by feedback), and if it fits the standard, the goal or aspiration has been met. In other words, responsibility has successfully been taken. If this is not the case, further activity will be done to achieve the goal of responsibility. Since responsibility is about achieving success or safeguarding against failure within a valued domain, responsibility incorporates motivation. From a hedonistic perspective, successful, responsible actions motivate due to positive emotions, as do avoiding negative emotions arising from failure. As mentioned earlier, scholars assume responsibility for failure to be a key mechanism in blame and guilt (Diamond and Allcorn, 1984; Weiner, 1995a, 1995b), while others focus on responsibility for success as sources of positive emotions such as pride and satisfaction (Hackman and Oldham, 1976). Thus, there are both promotive and preventive goals involved in responsibility, though the perception and emphasis on either avoiding failure and blame or on successful, responsible actions may depend on both situational and personal factors (Lauermann and Karabenick, 2011). Another perspective on responsibility lies in the attractive self-image of being responsible.

### Being (ir)responsible: the self-involving motivational dimension

The self-involving dimension of responsibility means that responsibility is cognitively tied to a person’s self-concepts. In other words, responsibility is (also) something that “*one is*,” and not only a self-regulation standard that motivates one to goal-directed action. This notion has motivational and emotional implications. Kahn (1990) investigated how people can occupy and fill out roles with more or with less engagement. According to Kahn (1990), people vary in how much they employ and express themselves in their role or, vice versa, they detach and withdraw themselves when performing their role. Kahn denoted this way of expressing oneself or not as engagement: “Personal engagement is the simultaneous employment and expression of a person’s “preferred self” in task behaviours that promote connections to work and to others, personal presence (physical, cognitive, and emotional), and active, full role performances” (Kahn, 1990, p. 700). In the present article, we designate formal (and informal) roles as a form of objective responsibility imposed by the external structure on an organizational member. As we have already stated, such objective responsibilities can be accepted, regulated, and rejected subjectively. Personal self-internalized values, goals, and interests may form the choice to accept or reject responsibility for a given area. In other words, one’s own values and goals may determine the magnitude of importance that people will attribute to an objective responsibility area. Applying Kahn’s theory leads us to expect that if people express themselves as responsible subjects, they will be engaged in the action. Moreover, the more people see themselves to be responsible, the stronger they may express their self-internalized values when acting upon the perceived responsibility.

Self-employment and self-expression underlie similar constructs, such as flow, effort, or intrinsic motivation (Kahn, 1990), and thus, Kahn seems to perceive these constructs to be strongly related to engagement, if not the same. Below, we review literature that looks at responsibility and intrinsic motivation.

### Intrinsically motivated actions and responsibility

Intrinsic job motivation is a major predictor of wellbeing and successful performance at workplaces (Fishbach and Woolley, 2022). I/O Psychology scholars have taken an interest in the concept since humanistic management (McGregor, 1960) and Hertzberg et al. (1959) distinction between (intrinsic) motivators and (extrinsic) hygiene factors. Hackman and Oldham (1975) defined internal work motivation as the degree of self-motivation to perform the job effectively based on positive emotions when working effectively (p. 162). Intrinsic motivation is inherently bound to action. It is an action with a particular nature. As Fishbach and Woolley (2022) state: “[Intrinsic motivation] emerges from a perceptual fusion between an activity and the goal it serves. These are inseparable parts of the same entity, thus forming a unified Gestalt” (p. 341). The question is whether subjectively experienced responsibility may cause such intrinsically motivated action.

Hackman and Oldham (1976) started research on the relationship between responsibility and intrinsic motivation. Job Characteristics Theory proposes that job autonomy enhances experienced responsibility for outcomes of one’s job tasks and that this, in turn, would cause higher intrinsic job motivation (as well as job satisfaction and job performance) (Hackman and Oldham, 1976). In the meta-analytical evaluation of the theory, almost a decade later, Fried and Ferris (1987) noticed that only a handful or fewer studies had included responsibility (and the two other mediational variables of the model) and called for more research on this issue. A few existing studies have shown that feelings of responsibility for work outcomes correlated strongly with internal work motivation. More than another decade later, a meta-analysis by Behson et al. (2000) noticed that job design researchers still generally omitted the three mediational variables in their research. However, their research showed that felt responsibility for the work outcomes correlated quite well with internal work motivation, and this was replicated in a meta-analysis by Humphrey et al. (2007) that showed that responsibility did mediate between job autonomy and intrinsic job motivation. We may interpret this link between responsibility and intrinsic motivation as follows: when someone chooses to take on responsibility for a given task because they feel capable of doing so, the task becomes more interesting and engaging than tasks for which they do not experience responsibility.

*Proposition 5*: When people upregulate their responsibility based on high levels of perceived power and self-efficacy/competence, the resulting actions are perceived to be intrinsically motivating.

### High-level extrinsically motivated actions

Subjective responsibility may also potentially lead to other forms of motivated action that are based on intrinsic rewards. As suggested, internalized values and motives may also direct and regulate the degree of subjective responsibility. According to Organismic Integration Theory, a sub-theory of Self-Determination Theory, “identified” and “integrated” regulation are other agentic motivation forms stemming from realizing internalized goals and values. According to Ryan and Deci (2000), people “identify” with goals and values, i.e., recognizing values and goals to be important to the person, but the values and goals are only partially internalized and self-involving. In “integrated regulation,” values and goals motivate because they are fully integrated and acknowledged by the person. The integration process requires autonomy to freely determine if and (re-)formulate how values and goals congruently fit with oneself. A self-determined process of internalization creates the highest level of extrinsic motivation. It is extrinsic, because the actions are not motivating in themselves, but are means to a self-determined value or goal. Intrinsic motivation differs from this by being inherently satisfying. The action per se elicits positive states, such as joy, interest, and enthusiasm. Since choices to accept or reject responsibility are also proposed to be affected by internalized values and goals, the degree of internalization may imply that responsibility motivates actions either as “identified regulation” or as “integrated regulation.” Meta-analytic research proposes that the more internalized values and goals are within the self, the better the outcomes in terms of performance and wellbeing (Ryan et al., 2022). Thus, meta-analytic results show that integrated and identified regulated actions are related to the same positive psychological states as intrinsic motivation, such as, for example, positive affect, vitality, enjoyment as well as self-efficacy and self-esteem (Howard et al., 2021). These theoretical notions lead us to propose that responsibility is based on one’s own, internalized motives, the resulting responsible actions may be motivated as identified, or integrated. It follows that the degree of internalization plays a decisive role in the kind of motivated action.

*Proposition 6*: When subjective responsibility is based on internalized motives (values, goals, etc.), responsible actions may be motivated as “identified” (partially internalized) or “integrated motivation” (fully internalized).

## Discussion

In the following subsections, we discuss the integration of the propositions and their related implications, limitations, and suggestions for future studies.

### Integration

Our analysis integrates the somewhat scattered ideas about what responsibility is into a comprehensive definition as *the personal experience of feeling obligated to take initiative or to take precautions to ensure success and avoid errors concerning a specific valuable target.* In Table 1, we contrast subjective and objective responsibility.

**Figure 3:**
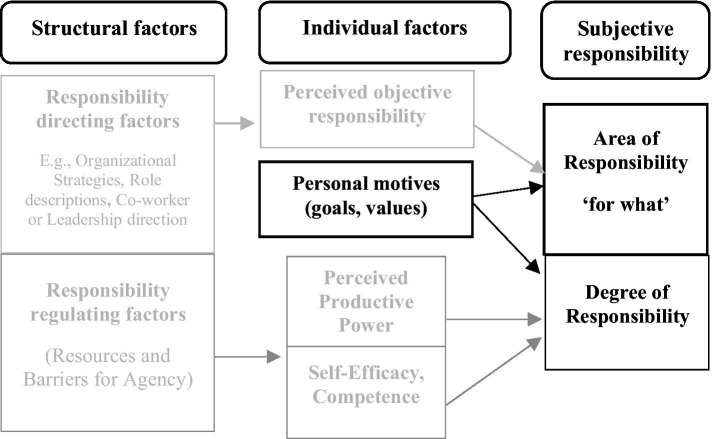
Personal motives and regulation of subjective responsibility. The figure extends Figure 2 by including how internalized personal motives may shape both the selection of what one feels responsible for and the extent to which one feels responsibility for it.

**Figure 4:**
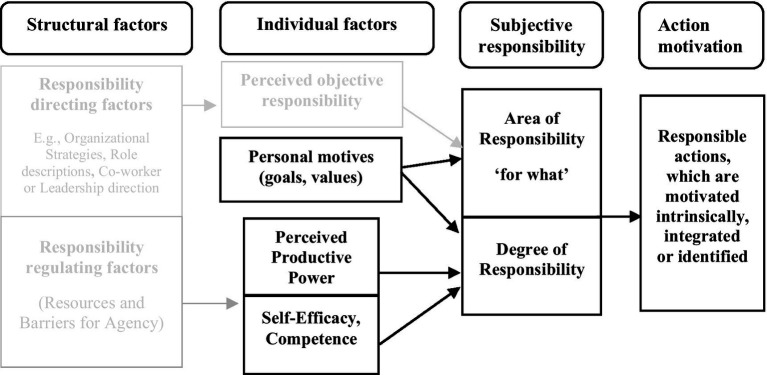
Subjective responsibility and action motivation types. The figure extends Figures 1–3 by adding a category about action motivation. Based on propositions 5 and 6, the figure shows how perceived power, self-efficacy, and internalized personal motives may shape an individual’s subjective responsibility—both in terms of what they feel responsible for and how strongly. This, in turn, may lead to responsible actions that are driven by intrinsic, identified or integrated forms of motivation.

**Figure 5:**
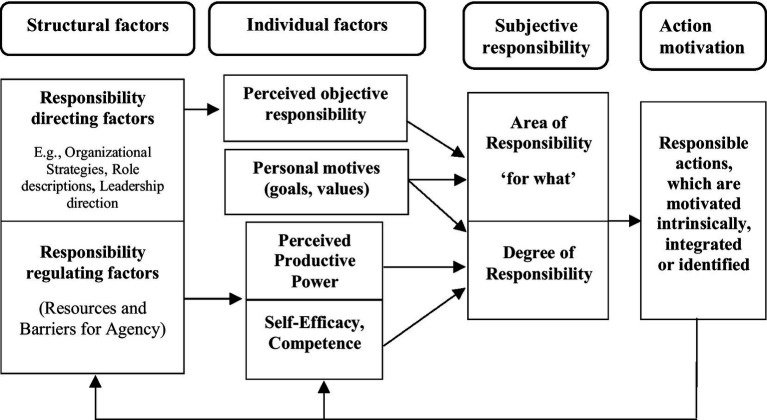
Integrated model of subjective responsibility in a structure-agency perspective. The figure integrates prior figures and illustrates the overall model developed throughout the article. Structural factors may influence individuals’ perceptions of what they are responsible for and their perceived capacity to act successfully. Combined with internalized personal motives, these perceptions shape how individuals subjectively regulate responsibility—both in terms of its content and intensity. In turn, subjective responsibility mediates how individual factors may cause responsible actions and how these actions are motivated, ranging from intrinsic to identified. Feedback arrows indicate that responsible actions may subsequently alter structural and personal properties in future cycles of subjective responsibility and actions.

We also used the limited extant research to develop theoretical propositions about the causes and consequences of subjective responsibility. The major notion here is that structural properties of an individual’s organizational environment both direct and facilitate (or hinder) what and how much the individual will experience responsibility. Since these work and organizational structural properties exist and operate independently of the individual, they can only affect the individual through how the individual perceives and interprets them. Thus, what one is responsible for in the job and organization, and the resources and barriers affect the individual through perceptions and an evaluation in terms of the individual’s own internalized values and motives. If the individual perceives himself or herself to have the power and self-efficacy/competence to act successfully and the responsible endeavor does not conflict with his or her own values, the individual will regulate their experience of responsibility to a high level. Depending on the relative weight of perceived power, self-efficacy/competence, and alignment with his or her own values and motives, the responsible action will take the form of intrinsic, integrated, or identified motivated actions. We integrate these theoretical propositions, which are depicted throughout the article in Figures 1–5.

### Implications

The conceptualization of subjective responsibility and the model also both have implications for future research. First, a clear definition may stimulate the development of hypotheses that would have been overlooked by using a narrower conceptual understanding. Second, the integrative definition may lead to the development of a more comprehensive measurement of the phenomenon that aims to cover all facets of experienced responsibility. The theoretical model can be applied to develop hypotheses within the different streams of organizational psychology research in responsibility, for example, micro-CSR (Glavas, 2016), responsible job performance (Hackman and Oldham, 1976; Pearce and Gregersen, 1991), or how responsible leadership influences employees to act responsibly (Pathak and Jha, 2024).

A theoretical implication of the model is that if responsibility is forced upon a person, that person may subjectively choose to downregulate or deny that responsibility. If external pressure leads to actions that are not aligned with the subjective regulation process, this would result in highly extrinsically motivating actions. According to Self-Determination Theory, this would lead to aversive consequences. Thus, in organizations, this kind of “forced responsibility” may be stressful, violate a person’s values and interests, and negatively impact the performance of these forced actions. A further, theoretical implication is that the different forms of motivated actions have been shown to come with several other consequences in terms of employee wellbeing, psychological development, and performance. As we deem these consequences to be implied and more distal, as well as more domain-specific, they have not been included in the present model.

To the extent that the proposed model can be supported empirically, the model suggests that organizational and leadership practices aimed at responsible organizational actions should consider how to strengthen individual agency and subjective responsibility. In practice, organizational empowerment and empowering leadership may be effective in combination with visionary directions of what is worth to be responsible for, if employees have corresponding values and motives.

### Limitations

The literature searches aimed to identify theoretical research about subjective responsibility and to map key areas of responsibility research within organizational psychology and related organizational fields. While extensive, this search was not intended to be a systematic review or meta-analysis, which would typically involve a more exhaustive and methodical process for empirical analysis. Instead, our goal was to synthesize existing theoretical perspectives on subjective responsibility, specifically within the context of organizational psychology. Thus, the literature review should be seen as a means for a conceptual synthesis rather than a comprehensive empirical analysis *per se*.

Though much literature treats responsibility as a moral concept, our definition does not explicitly indicate that subjective responsibility is about ethics and morality. Though this may appear to be a limitation, we think that the moral connotation lies in specifically what responsibility is for. Thus, moral values and reasons may differ from one domain to another. For example, responsibility for producing effectively (Jackson et al., 1993) has another kind and perhaps strength of ethics compared to responsibility for the world’s climate (Punzo et al., 2019). The present definition may, thus, also encompass various forms and degrees of ethics. Further research may elucidate how moral psychology constructs, such as for example moral identity (Boegershausen et al., 2015) or moral disengagement (Bandura, 2002), may relate to subjective responsibility for moral issues. We may cautiously speculate that moral identity may facilitate acceptance of a high level of responsibility for morally important areas, and that moral disengagement may be involved in downregulation of responsibility.

We believe that it is a limitation of the integrative model that it includes only two overall levels of analysis, i.e., the organization and the individual. For organizational psychology, interpersonal, intra-, and intergroup phenomena are just as important. For example, social psychologist Weiner (1995b) has treated attributions of responsibility for failures, and this and other interpersonal mechanisms could find their way into future models of subjective responsibility in organizations. Another relevant area of development could be how teams negotiate and share subjective experiences of responsibility. Furthermore, the model may also be further extended to include cultural levels and cross-cultural effects.

For the sake of parsimony, the model’s outcomes end with the individual’s motivated actions. Thus, more distal outcomes at both individual and organizational levels are not included. Depending on the area of responsibility, these may include individuals’ wellbeing, commitment, or task performance. It may also be relevant to include how individuals’ actions have consequences for organizational goal achievement, human capital, and change capacity.

Finally, the model is generally highlighting positive psychological phenomena. Though it may explain the erosion of responsibility agency (see Card, 2005) and the aforementioned “forced responsibility,” it does not focus on destructive forms of irresponsibility. Thus, there may be a difference between “forced responsibility,” “lack of responsibility,” and “destructive irresponsibility.” Future research may include such concepts and may also consider if or when subjective responsibility may be “too much” and have negative consequences for individuals and organizations.

## Conclusion

We searched and reviewed existing literature about subjective responsibility from the Scopus and PsycINFO databases. Our search results identified different streams of research in responsibility. They focus on organizational level, structural forms of responsibility, which we perceive as objective responsibility within the context of organizational members. Moreover, the results showed that research on subjective responsibility in organizational psychology and related fields is scarce, especially with respect to theoretical groundwork on the subject. Therefore, and to the best of our knowledge, we are the first to develop a theoretical groundwork on subjective responsibility. Based on different definitions and uses of the concepts in the literature, we derive an integrative definition of subjective responsibility. Departing from this and an agency theory perspective, we develop a model of antecedents and consequences of subjective responsibility. In this model, we suggest how structural factors in organizational members’ context direct as well as facilitate (or hinder) the individual’s agency. In turn, we suggest that agentic psychological states together with personal values and motives form the regulations of what and how much the person will choose to feel responsible for. Finally, we propose that moderate and high levels of subjective responsibility will lead to intrinsically, integrated, or identified motivated actions. In line with structure-agency theory, we propose that the actions taken reinforce or change the structural and individual factors behind future subjective responsibility regulations. We consider this work as a steppingstone for future empirical studies and theory development about subjective responsibility. We believe that such research may become more topical in an era where responsibility across a variety of areas is increasingly demanded.

## Data Availability

The original contributions presented in the study are included in the article/Supplementary material, further inquiries can be directed to the corresponding author.
