# Supplementary material for: Theorizing subjective responsibility at work: an agentic approach
Source: Front Psychol. 2025 Jul 10;16:1548931. doi: 10.3389/fpsyg.2025.1548931 (PMC12287070; doi:10.3389/fpsyg.2025.1548931)
Supplement: Supplementary file 1 [file Table_1.docx]

# **Supplementary Material**

**Table 1**

| **Overview of the Literature Review Process** | |
| --- | --- |
| **Step** | **Action** |
| Defining the Scope and Goal of the Review | - The goal is to explore and map the existing theoretical research on subjective responsibility in organizational psychology. The review focuses on conceptual work rather than a systematic review. |
| Identification of Relevant Journals in Scopus | - Conduct a broad search within Scopus under the ‘psychology’ category, filtering for relevant articles related to responsibility in the workplace. - Evaluate journals based on their inclusion in the Scimago database under the Applied Psychology category. - From the 48 journals found in Scimago, 21 were deemed relevant by the authors (see Table 2 below). |
| Selection of Business Management Journals | - Expand the search to include business management journals using the Chartered Association of Business Schools (ABS) Journal Ranking List. Focus on journals in Organizational Studies, Work and Organizational Psychology, Business Ethics, and Management. Additionally, we included the Annual Review of Organizational Psychology and Organizational Behavior and Frontiers in Organizational Psychology, two high-impact journals in the field. - A total of 116 journals were deemed relevant (see Table 3 below). |
| Article Selection and Screening in Scopus | - Identify relevant articles in the selected journals, ensuring they address the theme of responsibility in organizational and work settings. 72 articles were identified in the *Applied Psychology* domain and 120 articles in the *Business Management* domain. - Articles are classified based on relevance (Yes, No, Maybe). After screening, 147 articles were excluded for not meeting the relevance criteria, leaving 25 articles deemed relevant (16 from applied psychology, 9 from business management). |
| Supplementary Search in PsycINFO | - Perform a supplementary search in PsycINFO, focusing on specific psychological terms like "felt responsibility" and "experienced responsibility", while refining the search to classifications within Applied Psychology related to organizational behavior and human resources. This search resulted in 47 articles, with 22 deemed relevant after screening. |
| Integration into Theoretical Framework | - Analyze and integrate the selected articles into the theoretical framework of the study, ensuring the findings contribute to the development of the conceptual model of subjective responsibility in organizational contexts. |

1. **Code for the initial literature search in Scopus**

( TITLE-ABS-KEY ( work OR organization* OR business OR manage* OR employee* OR leader* OR personnel OR occupation* ) AND TITLE ( responsib* ) AND NOT TITLE-ABS-KEY ( corporate AND social AND responsibility ) AND NOT TITLE-ABS-KEY ( social AND responsibility ) AND NOT TITLE-ABS-KEY ( accountability )

AND NOT TITLE-ABS-KEY ( corporate AND social AND environmental AND responsibility ) ) AND PUBYEAR > 1969 AND PUBYEAR < 2026 AND PUBYEAR > 1969 AND PUBYEAR < 2026 AND ( LIMIT-TO ( SRCTYPE,"j" ) OR LIMIT-TO ( SRCTYPE,"b" ) OR LIMIT-TO ( SRCTYPE,"p" ) OR LIMIT-TO ( SRCTYPE,"k" ) OR LIMIT-TO ( SRCTYPE,"d" ) ) AND ( LIMIT-TO ( PUBSTAGE,"final" ) ) AND ( LIMIT-TO ( SUBJAREA,"PSYC" ) ) AND ( LIMIT-TO ( DOCTYPE,"ar" ) OR LIMIT-TO ( DOCTYPE,"ch" ) OR LIMIT-TO ( DOCTYPE,"re" ) OR LIMIT-TO ( DOCTYPE,"bk" ) ) AND ( LIMIT-TO ( LANGUAGE,"English" ) )

**Table 2**

List of Journal within the ‘applied psychology’ domain.

Out of the 160 journals initially identified in Scopus, 48 were found to be listed in Scimago under the *Applied Psychology* category and were considered relevant for our research. From these, we assessed each journal for relevance to the research question. After screening, 21 journals were selected, all of which were deemed directly relevant to the study of responsibility at work, specifically within the realms of work psychology and organizational behavior

| **Journal Title** | |
| --- | --- |
| 1 | Annual Review Of Organizational Psychology And Organizational Behavior |
| 2 | Applied Psychology |
| 3 | Assessment |
| 4 | Basic And Applied Social Psychology |
| 5 | Group And Organization Management |
| 6 | Human Resource Management |
| 7 | Humanistic Psychologist |
| 8 | Journal Of Applied Behavioral Science |
| 9 | Journal Of Applied Psychology |
| 10 | Journal Of Business And Psychology |
| 11 | Journal Of Career Development |
| 12 | Journal Of Managerial Psychology |
| 13 | Journal Of Occupational And Organizational Psychology |
| 14 | Journal Of Occupational Health Psychology |
| 15 | Journal Of Organizational Behavior |
| 16 | Journal Of Organizational Behavior Management |
| 17 | Journal Of Vocational Behavior |
| 18 | Organizational Behavior And Human Decision Processes |
| 19 | Organizational Dynamics |
| 20 | Personnel Review |
| 21 | Scandinavian Journal Of Management |

**Table 3**

List of Journals within the ‘business management’ domain included in the Literature Search in Scopus.

| **Journal Title** | |
| --- | --- |
| 1 | Organizational Science |
| 2 | Human Relationsa |
| 3 | Leadership Quarterly |
| 4 | Organization Studies |
| 5 | Organizational Research Methods |
| 6 | Group and Organization Management |
| 7 | Organization |
| 8 | Organization and Environment |
| 9 | Organizational Dynamics |
| 10 | Research in Organizational Behavior |
| 11 | Research in the Sociology of Organizations |
| 12 | Culture and Organization |
| 13 | Group Processes and Intergroup Relations |
| 14 | Journal of Co-operative Organization and Management |
| 15 | Journal of Organizational Behavior Management |
| 16 | Journal of Organizational Change Management |
| 17 | Journal of Professions and Organization |
| 18 | Leadership |
| 19 | Management Communication Quarterly |
| 20 | Negotiation Journal |
| 21 | Symbolic Interaction |
| 22 | Systemic Practice and Action |
| 23 | The Journal of Applied Behavioral Science |
| 24 | Action Research |
| 25 | Computational and Mathematical Organization Theory |
| 26 | Ephemera: Critical Dialogues on Organization |
| 27 | International Journal of Knowledge Management Studies |
| 28 | International Journal of Organization Theory and Behavior |
| 29 | International Journal of Organizational Analysis |
| 30 | International Journal of Project Organisation and Management |
| 31 | Journal of Organizational Ethnography |
| 32 | Journal of Applied Psychology |
| 33 | Personnel Psychology |
| 34 | Journal of Occupational and Organizational Psychology |
| 35 | Journal of Occupational Health Psychology |
| 36 | Journal of Organizational Behavior |
| 37 | Journal of Vocational Behavior |
| 38 | Organizational Behavior and Human Decision Processes |
| 39 | Work and Stress |
| 40 | Accident Analysis and Prevention |
| 41 | Applied Ergonomics |
| 42 | Applied Psychology |
| 43 | Ergonomics |
| 44 | European Journal of Work and Organizational Psychology |
| 45 | Human Factors |
| 46 | Human Performance |
| 47 | International Journal of Rehabilitation Research |
| 48 | Journal of Business and Psychology |
| 49 | Journal of Managerial Psychology |
| 50 | Journal of School Psychology |
| 51 | Occupational and Environmental Medicine |
| 52 | Scandinavian Journal of Work Environment and Health |
| 53 | Applied Psychological Measurement |
| 54 | Applied Psychology: Health and Well-Being |
| 55 | Cognition, Technology and Work |
| 56 | Group Dynamics |
| 57 | International Archives of Occupational and Environmental Health |
| 58 | International Journal of Industrial Ergonomics |
| 59 | International Journal of Selection and Assessment |
| 60 | International Journal of Stress Management |
| 61 | Journal of Applied Social Psychology |
| 62 | Journal of Career Assessment |
| 63 | Journal of Occupational Rehabilitation |
| 64 | Journal of Personnel Psychology |
| 65 | Journal of Safety Research |
| 66 | Organizational Psychology Review |
| 67 | Community, Work and Family |
| 68 | Disability and Rehabilitation |
| 69 | European Journal of Work and Organizational Psychology in Practice |
| 70 | Gedrag en Organisatie |
| 71 | Human Factors and Ergonomics in Manufacturing |
| 72 | Industrial and Organizational Psychology |
| 73 | International Coaching Psychology Review |
| 74 | International Journal for Educational and Vocational Guidance |
| 75 | International Journal of Occupational Safety and Ergonomics |
| 76 | International Journal of Workplace Health Management |
| 77 | International Negotiation |
| 78 | Journal of Career Development |
| 79 | Journal of Employment Counseling |
| 80 | Journal of Rehabilitation |
| 81 | Military Psychology |
| 82 | Occupational Medicine |
| 83 | Psychologie du Travail et des Organisations |
| 84 | Psychologist-Manager Journal |
| 85 | Rehabilitation Counseling Bulletin |
| 86 | Research in Occupational Stress and Well-Being |
| 87 | Revue Europeenne de Psychologie Appliquee |
| 88 | Social Work in Public Health |
| 89 | South African Journal of Industrial Psychology |
| 90 | Sport, Exercise, Performance Psychology |
| 91 | The International Journal of Aerospace Psychology |
| 92 | TPM – Testing, Psychometrics, Methodology in Applied Psychology |
| 93 | Work |
| 94 | Zeitschrift für Arbeits- und Organisationspsychologie |
| 95 | Annual Review of Organizational Psychology and Organizational Behavior |
| 96 | Academy of Management Annals |
| 97 | Academy of Management Journal |
| 98 | Academy of Management Review |
| 99 | Academy of Management Perspectives |
| 100 | Academy of Management Discoveries |
| 101 | Journal of Management |
| 102 | Administrative Science Quarterly |
| 103 | British Journal of Management |
| 104 | Business and Society |
| 105 | Business Ethics Quarterly |
| 106 | California Management Review |
| 107 | European Management Review |
| 108 | Gender and Society |
| 109 | Gender, Work and Organization |
| 110 | Harvard Business Review |
| 111 | International Journal of Management Reviews |
| 112 | Journal of Business Ethics |
| 113 | Journal of Business Research |
| 114 | Journal of Management Inquiry |
| 115 | MIT Sloan Management Review |
| 116 | Frontiers in Organizational Psychology |

1. **Code for the literature search in PsychINFO**

((tiab(work OR organization* OR business OR manage* OR employee* OR leader* OR personnel OR occupation*) NOT tiab(corporate social responsibility) NOT tiab(social responsibility) NOT tiab(accountability) NOT tiab(corporate social environmental responsibility)) NOT tiab(corporate) AND title(felt responsibility) OR title(experienced responsibility) OR title(subjective responsibility) OR title(sense of responsibility) AND cl(("Professional Impairment" OR "Organizational Psychology & Human Resources" OR "Personnel Evaluation & Job Performance" OR "Professional Personnel Attitudes & Characteristics" OR "Professional Psychological & Health Personnel Issues" OR "Industrial & Organizational Psychology" OR "Professional Ethics & Standards & Liability" OR "Personnel Management & Selection & Training" OR "Organizational Behavior" OR "Occupational Interests & Guidance" OR "Occupational & Employment Testing" OR "Management & Management Training" OR "Working Conditions & Industrial Safety" OR "Personnel Attitudes & Job Satisfaction")))

**Table 4**

List of Classifications Used in the Literature Search in PsycINFO Database.

| 1 | Professional Impairment |
| --- | --- |
| 2 | Organizational Psychology & Human Resources |
| 3 | Personnel Evaluation & Job Performance |
| 4 | Professional Personnel Attitudes & Characteristics |
| 5 | Professional Psychological & Health Personnel Issues |
| 6 | Industrial & Organizational Psychology |
| 7 | Professional Ethics & Standards & Liability |
| 8 | Personnel Management & Selection & Training |
| 9 | Organizational Behavior |
| 10 | Occupational Interests & Guidance |
| 11 | Occupational & Employment Testing |
| 12 | Management & Management Training |
| 13 | Working Conditions & Industrial Safety |
| 14 | Personnel Attitudes & Job Satisfaction |
